# Pharmaceutical Clinical Educators: A Resource for Advanced Practice Providers

**Published:** 2018-01-01

**Authors:** Sheila Adams Arrington, Laurie A. Farrell, Joanne Henning

**Affiliations:** 1 Genentech, a Member of the Roche Group, Germanton, North Carolina;; 2 Genentech, a Member of the Roche Group, Kissimmee, Florida;; 3 Genentech, a Member of the Roche Group, Collegeville, Pennsylvania

## Abstract

One of the main challenges within clinical practice today involves attaining the knowledge necessary to treat patients safely and effectively. The explosion of scientific breakthroughs within the health-care setting has created a new challenge for today’s practitioners: staying informed. In turn, the pharmaceutical industry has been challenged with providing information that is accurate, meaningful, and compliant with US Food and Drug Administration guidelines. In this article, we review how the pharmaceutical industry has tried to fill this need through the role of the pharmaceutical clinical educator (PCE). We describe the PCE role and the different forms of education and support that can be provided to advanced practice providers (APPs). We also address the conflict of interest issues surrounding a collaborative relationship between APPs and pharmaceutical industry APPs.

Over the past several decades, there have been significant scientific breakthroughs within the medical field, especially in the hematology and oncology community (Shelley, 2009; Wujcik, 2016). These breakthroughs include the awareness of new genetic markers, the development of targeted therapies, and other novel approaches to treating cancers such as altering our immune system (Wujcik, 2016). Advanced practice providers (APPs) need to commit to staying current with these advancements, but this can be a daunting task. Advanced practice providers need succinct yet accurate information in order to process and stay on top of the influx of changes in the oncology setting. Wujcik (2016) points out that nurses need to seek educational opportunities through professional organizations, the internet, and additional formal education. This is also true for APPs. Pharmaceutical clinical educators (PCEs) can be an additional resource for formal education. Despite concerns about the conflict of interest between pharma education and promotion, health-care providers (HCPs) feel there is some benefit between pharmaceutical industry and HCP interactions (Grundy, Bero, & Malone, 2013; Grundy, Bero, & Malone, 2016; Rashid, 2013). These benefits include education on both "branded" and "nonbranded" topics.

## WHAT IS A PHARMACEUTICAL CLINICAL EDUCATOR?

Many pharmaceutical and biotechnology companies employ clinical educators. Some companies, such as Genentech, label the PCE role "clinical coordinator," while other companies, such as Takeda, use "clinical nurse educator." For consistency, the term PCE will be used in this article. Aside from the title difference, it should be noted that this role varies from company to company. Some companies allow their PCEs to "develop responses and answer questions from healthcare providers and patients about the company’s products" (Ogbru, 2012, p 2), whereas other companies place restrictions around educating peers and patients. Lastly, it is the authors’ experience that the concept of a PCE within the pharmaceutical industry is a fairly new idea, with this role only being developed within the past 2 to 3 decades. Due to these factors, the PCE role, a description of the role, and a framework in which the PCE works, is hard to find in the literature.

The roles of a sales representative and a PCE within the pharmaceutical industry may appear very similar to APPs, yet there are several differences. The similarities and differences depend on with which division of the pharmaceutical company the PCE is aligned. A PCE who is aligned with the medical business can discuss off-label usage of a product or a medication currently in clinical trials. A PCE who is aligned with the sales representative in the commercial business division can never discuss off-label usage of a drug, or the status of clinical trial medications. These guidelines were put in place by the US Food and Drug Administration’s Office of Prescription Drug Promotion (FDA-OPDP). With that said, there are still distinct differences between a sales representative and a PCE. The main role of a sales representative is promoting a product and disseminating promotional information within FDA guidelines. A sales representative is hired for his/her sales expertise and experience in the pharmaceutical industry. On the other hand, the main role of a PCE is to educate APPs and other HCPs on a product’s safety and efficacy, dosing and administration, and often on disease states. Candidates for the role of a PCE in most pharmaceutical companies need to have a medical background, degree, license, and credentials within the health-care industry. A PCE is hired based on his/her clinical experience within the medical field, usually in a specific area of expertise. Due to the experience of PCEs, they can engage in meaningful peer-to-peer discussions with medical professionals and provide pertinent clinical information needed by all HCPs, including APPs.

Many pharmaceutical companies have realized the positive impact of peer-to-peer interactions and education and have embraced the role of the PCE (Shelley, 2009). Pharmaceutical clinical educators also play a significant role within industry due to their experience and knowledge of what APPs want and need to know about a particular product. With their in-depth knowledge, they can be a resource for the marketing team to help develop pertinent and FDA regulation-compliant slide presentations.

## EDUCATIONAL RESOURCES OFFERED BY PCES

Pharmaceutical clinical educators can be a significant resource to all APPs, providing both "branded" and "nonbranded" education. "Branded" education is defined as any education that includes the name of a product (i.e., drug specific; Shelley, 2009). This type of education ensures that APPs have accurate, relevant information on treatment options that are FDA approved and included in the manufacturer’s package insert. Advanced practice providers are educated to understand and be able to clearly articulate to the patient what to expect throughout their therapy as well as why this treatment option is being offered (Robinson, 2016). This is one of the most important ways that pharmaceutical companies can support APPs and ultimately impact the lives of patients. Needless to say, this type of information is highly regulated and must comply with the FDA-OPDP. All prescription drug information must be truthful, balanced, and accurately communicated. This is accomplished through a comprehensive surveillance, enforcement, and education program (Baker, 2012).

"Nonbranded" education is defined as educational information that is absent of any product or is not specific to any company (Shelley, 2009). An example of nonbranded education would be something that is related to patient care, such as infusion-related reactions. Pharmaceutical clinical educators may have been involved in managing and treating patients experiencing infusion-related reactions while in the clinic. They have firsthand experience in managing patient reactions and mediating mechanisms of action based on the prescribing information. Because of their experience and educational background, PCEs can present in-depth information on symptom management in addition to disease states. Baker (2012) reported a bold upswing in the use of nonbranded material in 2009. The Pharmaceutical Research and Manufacturers of America (PhRMA) set up a voluntary policy to task pharmaceutical companies to become more of a resource to HCPs. This challenged the pharmaceutical companies to tell a "deeper more compelling story to both patients and HCP" (Baker, 2012). This initiative has spurred the development of more effective tools to educate patients and APPs.

## IMPACT ON INDUSTRY

Pharmaceutical clinical educators serve as a resource for senior leadership, marketing, and sales in the pharmaceutical industry by providing authenticity to their interactions with APPs. Their wealth of clinical experience and knowledge within the health-care field can provide industry leaders with key insights into which resources, tools, and education are needed by APPs to provide the best care possible for their patients and their families.

## ETHICAL CONCERNS

Over time, with the development of the role of the PCE, there has been an influx of registered nurses, nurse practitioners, PAs, and pharmacists entering the pharmaceutical industry. Health-care providers in the role of PCEs most likely had a past experience interacting with pharmaceutical representatives and are aware of the conflicts of interest and ethical concerns that can arise from these interactions, such as commercial biases in the education, the risk for payment for speaker engagements being perceived as payoffs for prescribing, and completely false product information (Grundy et al., 2013; Grundy et al., 2016; Ladd, 2011; Pizzo, Lawley, & Rubenstein, 2017; Rashid, 2013). As former clinicians and now PCEs, the authors’ main goal is to provide reliable, factual information that is unbiased. Shelley (2009) points out that "educational messaging may be skewed (intentionally or unintentionally) as a result of the [nurse educators’] direct link to the pharma companies underwriting the training program and supplying the information" (p 7). She also commented on "transparency as being the key to maintaining integrity and trust" between PCEs and APPs (Shelley, 2009, p 8). There is an appreciation that a fine line exists between education and promotion. It is these authors’ opinion that the oath taken as HCPs for licensure to do no harm, the intent to educate, and the FDA guidelines distinguish educational presentations from sales representative promotional presentations.

Clearly, the ethical concerns concerning the relationship between the pharmaceutical industry and APPs are a double-edged sword. Both parties can either benefit or be negatively affected by the debate. A study done by Grundy and colleagues (2016) showed 16 out of 56 nurses interviewed reported that it would be "impossible to do their jobs without industry resources" (p 735). Meanwhile, facilities have denied access to pharmaceutical representatives, both sales representatives and PCEs, to prevent any impression of conflicts of interest (Robinson, 2016). Advanced practice providers request information, whether promotional or nonpromotional, because they have identified a need for themselves or their caregivers, yet access to these resources for APPs can be restricted.

Unfortunately, perceived and actual conflicts of interest cannot be avoided and there is a need to combat these concerns. Pizzo, Lawley, and Rubenstein (2017) suggested having leaders of academic medical centers (AMCs) play a part in this process. By creating boundaries with the way the pharmaceutical industry and HCPs, including APPs, collaborate, there is a way both parties can benefit. We believe an increase in collaboration between policy makers within AMCs and policy makers within pharmaceutical companies is a starting point for setting these boundaries.

Another way to combat this concern is to educate oneself. Several suggestions have been made in the literature. Rashid (2013) offered a perspective on how to distinguish between marketing and education. He pointed out that the use of evidence-based medicine can help reconcile the difference between both promotional and nonpromotional education, yet the science of epidemiology and biostatistics is not well taught or understood by many. This idealism essentially places the onus on the APP to determine the difference between promotion and education. Most, if not all APP degrees now require a class in evidence-based practice, so one could challenge Rashid’s opinion that APPs are not equipped to distinguish between marketing and education (Hande, Williams, Robbins, & Christenbery, 2017). Gleason and Schaffer (2013) took this idea a step further and offered up the protocol STEPS, which is a mnemonic for safety, tolerability, effectiveness, price, and simplicity. They claim this mnemonic can be used to distinguish the difference between promotional and nonpromotional or evidence-based data. Ladd (2011) suggested self-reflection and examination of personal philosophy in separating promotional from nonpromotional education. Other suggestions, such as creating guidelines and considering specific situations and discussing with peers have been proposed (Crigger, 2005). Regardless of the suggestion APPs choose to follow, increasing knowledge and understanding evidence-based medicine will only help improve collaboration and patient outcomes.

## CONCLUSION

The job description and impact of a PCE far exceeds those that are highlighted in this article. The purpose of this Practice Matters feature is to highlight the benefits that result from a collaborative relationship between APPs and APP colleagues who work in the pharmaceutical industry as PCEs. Patient outcomes can be affected by having knowledge of up-to-date, pertinent data. A collaborative relationship between APPs and PCEs can help increase this knowledge, and ultimately improve patient and population outcomes. It is important to be aware of the concerns with conflicts of interest and ethical implications, but it is equally important to educate oneself to impact the care we provide. More research and literature is needed to distinguish the role of the PCE from the sales representative, and to create a framework for the role of the PCE within the pharmaceutical industry. Additionally, as clinicians, we have taken an oath to care for patients and we need to continue to foster peer-to-peer collaboration to improve health-care outcomes.
